# Evolution Strategies in Transaxillary Robotic Thyroidectomy: Considerations on the First 449 Cases Performed

**DOI:** 10.1089/lap.2019.0021

**Published:** 2019-04-10

**Authors:** Micaela Piccoli, Barbara Mullineris, Davide Gozzo, Giovanni Colli, Francesca Pecchini, Casimiro Nigro, Vincenzo Rochira

**Affiliations:** ^1^ Department of General and Emergency Surgery, Azienda Ospedaliero-Universitaria Modena, Modena, Italy.; ^2^ General Surgery Department, Torvergata University, Rome, Italy.; ^3^ Azienda Ospedaliero-Universitaria di Modena, Modena, Italy.; ^4^ Unit of Endocrinology, Department of Biomedical, Metabolic and Neural Sciences, University of Modena and Reggio Emilia, Modena, Italy.

**Keywords:** robotic, thyroidectomy, Modena retractor, transaxillary

## Abstract

***Background:*** In the past 20 years, the fast spread of new surgical technologies has reached an important peak with the advent of the robotic surgery. Many studies have been run about a cosmetic desire to avoid neck scars after thyroid surgery and this has led to the development of remote access robotic thyroidectomy (RT). Among the various RT approaches, unilateral transaxillary access is one of the most widely used, reporting excellent results in terms of feasibility and patient's compliance. The mini-invasive technique demonstrated many potential shortcoming overcomes with the robotic approach. At our institution a team of 3 skilled endocrine surgeons with experience in laparoscopic and robotic procedures performed RT. Our aim is to report our 8-year single-centre robot-assisted thyroidectomy experience, by applying a gasless unilateral transaxillary approach with the so-called hybrid technique, and to demonstrate its safety and feasibility.

***Methods:*** In the period between September 2010 and June 2018 at our institution, a total of 472 patients underwent thyroid and parathyroid transaxillary surgery. The hybrid technique was applied for all the robotic procedures. A total of 412 procedures were performed with the use of external “Modena Retractor” (CEATEC^®^ Medizintechnik) and with 3 surgeons. According to international guidelines, our indications for robotic surgery were benign lesions with a diameter <5 cm, Graves' disease, well-differentiated thyroid cancers, and parathyroid adenomas.

***Results:*** In this series, a total of 449 cases were registered. General data of patients were analyzed: gender, age, body mass index, tumor size, preoperative fine-needle aspiration examination, definitive histological examination, operative time, and postoperative complications.

***Conclusions:*** This study confirms the application of robotic approach in thyroid surgery as a feasible technique in terms of safety and complications risk. The hybrid technique, together with a dedicated surgical team, can lead to obtaining the same outcomes of traditional anterior cervicotomic surgery, adding a scarless thyroidectomy.

## Introduction

In the past 20 years, the fast spread of new surgical technologies has reached an important peak with the advent of the robotic surgery.^1^ This new surgical technique extended its target to many specialties. The interest of producers, associated with the desire of a “scarless surgery,” leads the main centers to concentrate their efforts on it.^2^ As for any innovations, surgeons have been studying and learning how to manage the new device, planning all the operation steps, and acquiring the necessary skills to perform the procedures in safety. Many studies have been run about a cosmetic desire to avoid neck scars after thyroid surgery and this has led to the development of remote access robotic thyroidectomy (RT).^3,4^

Among the various RT approaches, unilateral transaxillary access is one of the most widely used,^5^ reporting excellent results in terms of feasibility and patient's compliance.^6,7^ The mini-invasive technique demonstrated many potential shortcoming overcomes with the robotic approach.^8^ At our institution a team of 3 skilled endocrine surgeons with experience in laparoscopic and robotic procedures performed RT. Our aim is to report our 8-year single-centre robot-assisted thyroidectomy experience, by applying a gasless unilateral transaxillary approach with the so-called hybrid technique,^9^ and to demonstrate its safety and feasibility.

## Materials and Methods

**FIG. 1.**
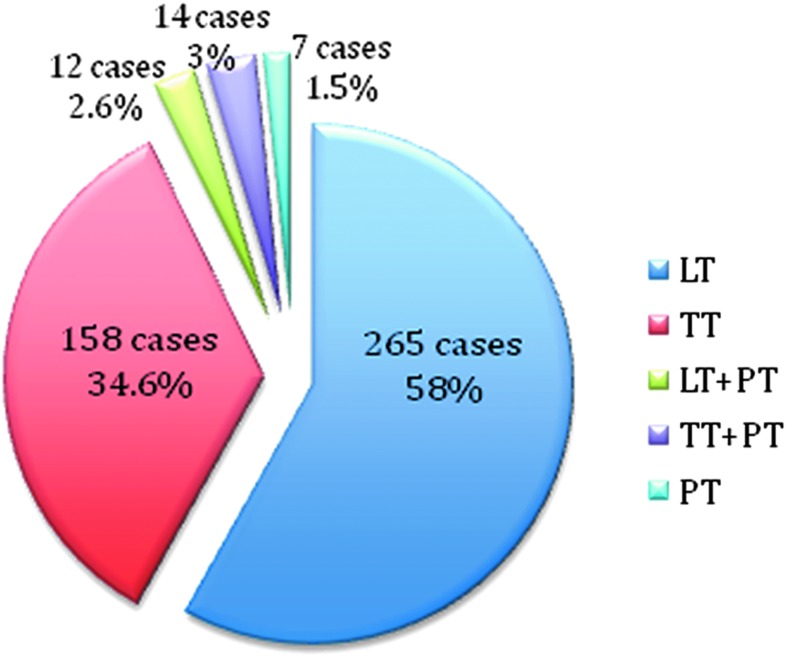
Total number of robotic procedures. LT, hemithyroidectomy; PT, parathyroidectomy; TT, total thyroidectomy.

In the period between September 2010 and June 2018 at our institution, a total of 472 patients underwent thyroid and parathyroid transaxillary surgery; among these a total of 16 patients were treated with unilateral transaxillary endoscopic technique, whereas for 456 patients the robotic procedure was performed. In particular we registered 158 cases of total thyroidectomy (TT), 265 cases of hemithyroidectomy (LT), 14 total thyroidectomy with target parathyroidectomy (TT+PT), 12 cases of hemithyroidectomy combined with parathyroidectomy (LT+PT), and 7 single parathyroidectomies (PTs) (Fig. 1). Excluding target PTs, we analyzed a total of 449 cases. The hybrid technique was applied for all the robotic procedures: endoscopic approach for the flap time and robotic one for the console time. In total 52 operations were performed using da Vinci^®^ S surgical robotic system (Intuitive Surgical, Sunnyvale, CA) and 397 with da Vinci Si surgical robotic system. At the beginning of our experience, 37 procedures were performed with involvement of 4 surgeons: the first operator dedicated both to the working space and the console time, the second surgeon handled the camera, while the third and fourth operators kept the manual external retractor until the console time started with “Chung Retractor” positioning. A total of 412 procedures were performed with the use of external “Modena Retractor” (CEATEC^®^ Medizintechnik) and with 3 surgeons.

According to international guidelines, our indications for robotic surgery were benign lesions with a diameter <5 cm, Graves' disease, well-differentiated thyroid cancers, and parathyroid adenomas. We gave contraindications to robotic approach in case of previous neck or breast surgery, neck radiotherapy, pacemaker implant in the major pectoralis region in case of necessity of same side axillary access, shoulder arthrosis, and previous shoulder surgery. For each patient the following data were collected: age, gender, body mass index (BMI), tumor size, fine needle aspiration biopsy (FNAB) diagnosis, operation time (flap, docking, and console), total drain output, hospital length of stay, and postoperative complications (such as hypocalcemia, nerve injury, flap burnt, flap hematoma, and shoulder discomfort). Helsinki criteria were followed and all patients were required to express their informed consent before surgery. All data were collected in a database and statistically analyzed.

Before surgery, all patients underwent endocrine evaluation, neck ultrasonography imaging, and in case of necessity a fine-needle aspiration (FNA) examination was done; moreover, anesthesiologist visit, ECG, vocal cord evaluation, chest X-ray, and laboratory examinations, including thyroid function parameters were required.

### Surgical technique

As reported before, at the beginning of our experience 37 robot-assisted thyroidectomies were performed by adopting the Woon Youn Chung position and technique,^3^ with 4 surgeons involved. The endoscopic vision, with a camera and an external screen involvement, during the flap creation represents a difference from Chung's technique. All surgical team follows a direct flap creation, and laparoscopic instruments utilization reduces the axillary skin length incision. By the years, achieving more experience, we modified the Chung's arm position for the patient on the surgical table to reduce cervical brachial plexus injury and postoperative shoulder discomfort. Moreover, at the beginning the da Vinci S surgical robotic system required an 8 mm robotic anterior chest wall trocar placement, with the advent of the da Vinci Si surgical robotic system only a paraxillary skin incision trocar placement was then required.

### Patient position

Patient is placed in a supine position with a slight neck extension. The arm on the surgical access side is raised upon the head with a 90° angle elbow abduction to minimize the distance between the axilla and the neck region; arm position is checked, while the patient is awake before undergoing general anesthesia, to avoid wrong position and consequent brachial plexus injury. The contralateral arm is placed along the body. Another support is placed near the head to prevent any lateral movements during the procedures. Bladder catheter was positioned only at the beginning of the experience due to the longer operative time. The axillary access was chosen according to the side of the largest nodule or target well-differentiated thyroid carcinoma (for TT), thyroid lobe to remove (for lobectomy), and target PT.

### Robot position

The da Vinci Si robot is docked contralaterally to the axillary access. Three arms are used for lobectomy with <4 cm diameter lesions and for target PTs; four arms are needed for total thyroidectomies and for lobectomies with nodule >4 cm.

### Hybrid procedure

Step 1: Working space or flap creation time: Through a 4–5 cm axillary incision, under a 30° camera endoscopic vision standing over the anterior surface of the pectoralis major muscle, a subplatysmal skin flap from the axilla to the anterior neck area is performed by using laparoscopic instruments such as Johann grasper and monopolar hook.^9^ The endoscopic vision means a longer working time if compared with the direct view,^3^ but it ensures a more precise tissue dissection with a clearer anatomical landmarks visualization and less bleeding risk and strap muscle sparing. Moreover, we can assume that this technique, allowing the surgical team to follow the flap creation, reduces the learning curve. Before the procedure starting, an external retractor, the so-called Modena Retractor (CEATEC Medizintechnik),^9^ is positioned to create the flap: it offers important advantages in terms of better surgical field visualization with surgeon's tremor abolition and sparing human resources. The Modena retractor is a surgical table-fixed device, managed only by one surgeon and with the possibility to be connected to a suction tube to reduce surgical field fogging. During the procedure, the device can be repositioned by the surgeon at the operating table while the tunnel creation progressively goes on, and a constant traction of the flap is kept to obtain an easier tissue dissection. The aim of this time is to reach the sternocleidomastoid muscle and to dissect the sternal head (upside) and the clavicular head (downside) while the Modena retractor is repositioned beneath sternal head. Surgeons are required to be very careful during this step because overlying skin thermal burns are frequent and an inadequate traction with the Modena retractor could create an ischemic skin suffering and a subsequent skin necrosis. The omohyoid muscle, the superior landmark of the surgical field, has to be identified and dissected from the strap muscle. At this point, Modena retractor is replaced beneath the strap muscle and thyroid gland is reached. In case of TT, dissection continues to the contralateral strap muscle that are lifted up with the Modena retractor too.

Step 2: Docking time: Robot (da Vinci Si) is docked at the operative field. A further small incision is created at the inferior part of the axillary incision to insert a 5 mm robotic trocar for Maryland forceps or Harmonic scalpel introduction. At first, the da Vinci S surgical robotic system required an 8 mm robotic anterior chest wall trocar^3^; with the introduction of the da Vinci Si surgical robotic system only one additional 5 mm trocar is required with an inferior paraxillary access; this trocar access, added to the 4–5 cm axillary incision, reduces robotic arms conflict and it is also used for drainage positioning at the end of the procedure. For lobectomy, three robotic arms are positioned (two through the axillary skin incision and one through external one independent incision), while four robotic arms are used for TT (three inside the axillary skin incision and one from the independent one). A 30° robotic camera is positioned in the middle of the axillary access and the Maryland forceps and Harmonic scalpel could move from side to side.

Step 3: Console time: One surgeon stands at the operating table while the first operator moves to the robotic console. First, the upper pole of the thyroid is drawn downward and medially using the Prograsp or Maryland forceps; superior thyroid vessels are identified and individually divided close to the thyroid gland to avoid injury of the external branch of the superior laryngeal nerve, and the middle thyroid vein is then dissected. All vessels are dissected with Harmonic scalpel. The inferior thyroid artery (ITA), the recurrent laryngeal nerve (RLN), and the parathyroid glands are identified. ITA is then dissected close to the thyroid, and the whole lobe is separated from the trachea and removed with the isthmus, with attention to RLN preservation. The specimen is removed through the axillary incision. Surgeon at the table ensures the correct positioning of robotic arms and uses a suction-retractor device to keep the operative field clean and enlarged. If a TT is required, the contralateral lobe is dissected with a medial traction of the thyroid. Contralateral RLN identification represents the most challenging step of the procedure, and a gentle medial traction of the trachea with the external suction-retractor (performed by the surgeon at the table) is necessary to visualize the nerve and to avoid injuries. Once the RLN course is traced, the dissection can be achieved using the same method, and finally the contralateral lobe is removed. At the end of the procedure, the bleeding is checked with Valsalva maneuver and a suction drain is inserted through the external axillary incision. The wound is cosmetically closed with an absorbable running suture and the axilla scar results to be completely covered by the natural arm position.

## Results

**FIG. 2.**
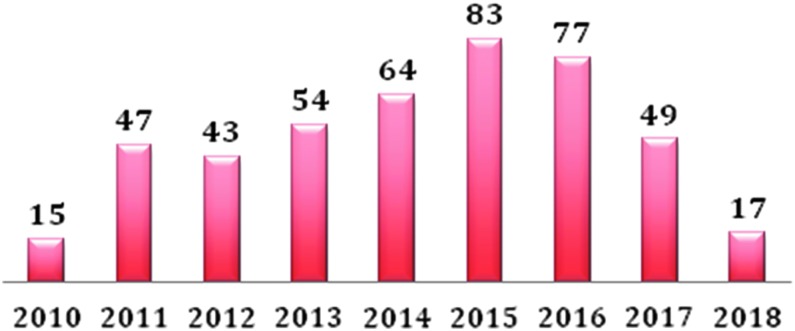
Number of robotic procedures by year.

**FIG. 3.**
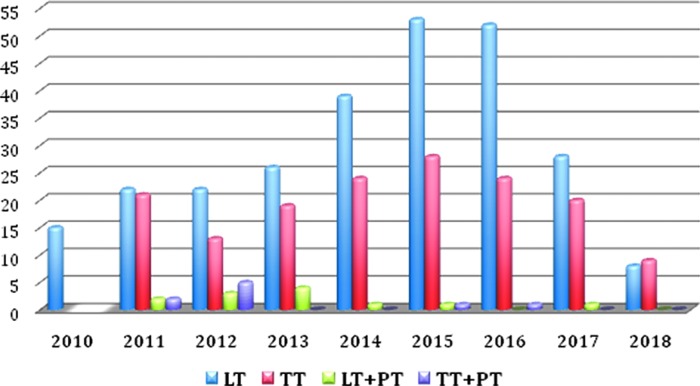
Number of robotic procedures by year and number of different robotic procedures by year. LT, hemithyroidectomy; PT, parathyroidectomy; TT, total thyroidectomy.

In this series, a total of 449 cases were registered; Figures 1–3, respectively, report the different robotic transaxillary procedures performed and the by year subdivision. General data of patients are summarized in Table 1: we registered 387 women and 62 men (F/M 86.1%/13.8%), mean age at the operation time was 44.1 ± 12.1 years; median BMI was 23.9 ± 3.9. Median tumor diameter was 26.6 mm (±13.1). At preoperative FNAB we had 12 Tir1, 85 Tir2, 267 Tir3, 11 Tir4, and 4 Tir5, 11 cases were classified as Plummer's adenoma, 4 as Hurtle cells adenoma, and 20 cases were Graves' disease, for 35 patients the FNAB was not performed. Of the total of 265 emithyroidectomies, 36 cases were radicalizations of malignant lesions resulted at the histological examination of previous surgery. At definitive histological examination we registered 335 cases (74.94%) of benign lesions and 112 (25.05%) of malignant ones (Table 2). Considering all the robotic procedures, median operative time was 117.5 ± 40.3; in particular for the working space (flap time) the median time was 53.8 ± 19.9 minutes, median docking time was 10.9 ± 6.9 minutes, and the console one was 52.5 ± 26.8 (Table 3). Median time for specific surgical procedures is reported in Table 4.

**Table 1.** General Data

Median age (years)	44.1 (±12.1)
Gender (M/F)	62/387 (13.8%/86.1%)
Median BMI (kg/m^2^)	23.9 (±3.9)
Median tumor size (mm)	26.6 (±13.1)

BMI, body mass index.

**Table 2.** Histological Data

	*N pts*	*%*
*FNAB*		
Tir1	12	2.67
Tir2	85	18.93
Tir3	267	59.46
Tir4	11	2.44
Tir5	4	0.89
Other	35	
Plummer	11	2.44
Hurtle	4	0.89
Basedow	20	4.45
Not performed	35	7.79
Totalization after LT	36	
Definitive histology		
hyperplasia	197	44.07
Follicular adenoma	74	16.55
Hurtle cell adenoma	18	4.02
PTC	98	21.92
FTC	7	1.56
MTC	1	0.22
No neoplasia	25	5.59
Associated parathyroid	27	6.04
Benign lesions	335	74.94
Malignant lesions	112	25.05

FNAB, fine needle aspiration biopsy; LT, hemithyroidectomy; Pts, patients.

**Table 3.** Surgical Procedures

	*N pts (%)*
Type of surgery	
LT	265 (59.02)
LT dx	137 (51.69)
LT sx	128 (48.3)
TT	158 (35.18)
LT+PT	12 (2.67)
LT dx	5 (41.66)
LT sx	7 (58.33)
TT+PT	14 (3.11)
Conversion to open	3 (0.66)
Median operation time (minutes)	117.5 (±40.3)
Flap time	53.8 (±19.9)
Docking time	10.9 (±6.9)
Console time	52.5 (±26.8)
Median hospital stay (days)	1.84 (±0.8)

dx, right; LT, hemithyroidectomy; LT+PT, hemithyroidectomy with target parathyroidectomy; PT, parathyroidectomy; sx, left; TT, total thyroidectomy; TT+PT, total thyroidectomy with target parathyroidectomy.

**Table 4.** Median Time for Different Surgical Procedures (Minutes)

*Type*	*Total*	*Working space*	*Docking*	*Console*
LT (*n* = 265)	103.6 (±35.8)	51.6 (±19.9)	10.2 (±7.1)	41.6 (±23.6)
TT (*n* = 158)	139.6 (±37.1)	56.5 (±19.6)	11.6 (±5.5)	70.8 (±22.2)
LT+PT (*n* = 12)	100.5 (±30.8)	53.08 (±16.4)	13.5 (±14.1)	33.8 (±10.1)
TT+PT (*n* = 14)	149.2 (±36.2)	64.5 (±18.6)	13.5 (±3.7)	71.1 (±19.3)

LT, hemithyroidectomy; LT+PT, hemithyroidectomy with target parathyroidectomy; PT, parathyroidectomy; TT, total thyroidectomy; TT+PT, total thyroidectomy with target parathyroidectomy.

Median hospital stay was 1.84 ± 0.8 days, serum calcium values was dosed in first and second postoperative days, and at least two specialistic outpatient postdimission controls were planned.

Table 5 shows postoperative complications: transient hypocalcemia was reported for 72 patients (16.03%), all treated with oral calcium therapy, and no cases of permanent hypocalcemia were described at follow-up. There were 4 cases of thyroid lodge bleeding (0.89%), 2 of which required conversion to traditional/open surgery; 8 patients presented flap hematoma (1.7%) and in 2 cases wound axillary incision for local hemostasis control was needed. Five patients presented wound seroma (1.5%) and skin burn occurred only in 1 case (0.22%). One patient experienced postoperative transient neck paresthesia (0.3%), 7 patients reported chest transient paresthesia (1.55%), and in 10 cases (2.22%) shoulder discomfort was described. Focusing on recurrent laryngeal nerve lesions, the most representative risk of thyroid surgery, we registered 17 patients (3.78%) presenting postoperative dysphonia; all patients underwent otorhinolaryngologist evaluation and monolateral recurrent laryngeal lesion was confirmed in 15 cases (9 lateral vocal cord palsies and 6 medial vocal cord palsies) and a specific logopedic therapy was then undertaken for 13 patients, whereas 2 people decided to call on another institute. At follow-up we can report a complete functional recovery for 12 patients and for 1 case a permanent medial cord palsy was confirmed.

**Table 5.** Postoperative Complications

	*Total,* n *(%)*	*LT,* n *(%)*	*TT,* n *(%)*	*LT+PT,* n *(%)*	*TT+PT,* n *(%)*
Hypocalcemia	72 (16)	15 (20.8)	52 (72.2)	1 (1.3)	4 (5.5)
Transient	72	15	52	1	4
Permanent	—	—	—	—	—
Dysphonia	17 (3.78)	6 (35.2)	10 (58.8)	1 (5.8)	—
Recurrent laryngeal nerve palsy	15 (3.3)	5 (33.3)	10 (66.6)	—	—
Transient	12	4	8	—	—
Permanent	1	—	1	—	—
Transient hoarseness	11 (2.44)	5 (45.4)	5 (45.4)	1 (9.09)	—
Bleeding	4 (0.89)	1 (25)	3 (75)	—	—
Wound seroma/infection	5 (1.5)	4 (80)	1 (20)	—	—
Flap hematoma	8 (1.7)	2 (25)	6 (75)	—	—
Skin burn	1 (0.22)	—	1	—	—
Neck paresthesia	1 (0.3)	1	—	—	—
Chest paresthesia	7 (1.55)	5 (71.4)	2 (28.5)	—	—
Shoulder discomfort	10 (2.22)	8 (80)	2 (20)	—	—

LT, hemithyroidectomy; LT+PT, hemithyroidectomy with target parathyroidectomy; TT, total thyroidectomy; TT+PT, total thyroidectomy with target parathyroidectomy.

## Discussion

In the past two decades, robotic-assisted transaxillary thyroid and parathyroid surgery has widely spread, according to the high technological progress and the increasing request by patients, with the aim to get best clinical, aesthetic, and technological results.^10–12^ Southeast Asian surgeons have been first involved in this new kind of surgery in consideration of patient's desire to avoid neck scars, most of them young women with religious issues.^13^ The advent of minimally invasive and remote access approach for thyroid surgery was in 1997 with the first endoscopic thyroidectomy,^14^ while the first RT using the da Vinci System was first performed by Chung in 2007.^15^ After the first Korean robotic transaxillary scientific publications, some American surgeons strongly fought the spread of this technique, pointing out the expensive costs of the procedure, the long and difficult surgeons training, and the few numbers of thyroid procedures performed per year in a single surgical unit.^16^ Moreover, the da Vinci manufacturer Intuitive Surgical, on October 2011, disseminated an electronic notice that stated: “There will be no promotional material, no education, no assistance with proctors, no training course, and no procedural backup for thyroid resection.”^17^

Many studies have then described the advantages of robotic procedures in terms of safety, precision, tremor-free, three-dimensional view, and imaging magnification; da Vinci system helps to overcome some limitations of both traditional and endoscopic procedures,^18–20^ ensuring comparable oncologic outcomes.^21^ According to recent statement reported by American Thyroid Association, the remote-access thyroidectomy may be performed safely only in high-volume centers for well-selected patients undergoing strict selection criteria.^22^

Nowadays, literature confirms the feasibility of this procedure, with acceptable outcomes in terms of complications.^23,24^ In our series, 16% of patients had postoperative transient hypocalcemia, then gradually resolved with oral calcium intake; 2.44% had transient hoarseness, and only 3.3% of the patients presented vocal cord palsy requiring logopedy. Only 2 patients required a cervicotomy to control postoperative bleeding, otherwise conservatively managed, and 8 patients experienced neck or chest paresthesia, gradually resolved in few days.

Considering the median operation time occurred for the four different surgical groups, hemithyroidectomy (LT), hemithyroidectomy with target parathyroidectomy (LT+PT), TT and TT+PT, we saw that LT and LT+PT groups required less operative time both than TT and TT+PT (*P* < .05). No time differences comparing, respectively, LT with LT+PT and TT with TT+PT were reported, demonstrating that target PT in combination with robotic thyroid surgery does not require longer surgical time (Table 6). Moreover, Table 6 shows that, as expected, console time reflects operative time with the same statistical significative data (*P* < .05) when comparing the four groups.

**Table 6.** Median Time for Different Surgical Procedures (Minutes)

	*Operative time*		*Console time*	
*Type*	*SE*	P	*SE*	P
LT < TT	13.032	.000	13.022	.000
LT < TT+PT	35.413	.000	35.386	.000
LT+PT < TT	38.686	.001	38.656	.000
LT+PT < TT+PT	50.802	.002	50.763	.000
LT+PT > LT	38.113	.862	38.084	.333
TT < TT+PT	36.029	.425	36.001	.844

LT, hemithyroidectomy; LT+PT, hemithyroidectomy with target parathyroidectomy; SE, standard error; TT, total thyroidectomy; TT+PT, total thyroidectomy with target parathyroidectomy.

According to literature, our mean total surgical time results to be inferior to other European series^25^: this data could be explained by the high number of patients enrolled at our institution and the progressive derived experience reached by the surgeons.

We had a predominance of benign lesions (335 cases), with a 25% resulted as malignant carcinoma. Comparing the median console time required in case of benign and malignant lesions, respectively, of 51.27 and 56.45 minutes, we noticed a slightly higher duration for malignant ones with a higher operation time related to tumor diameter increasing; no remarkable differences in terms of complications rate were registered between the two groups. These data support us to strengthen the indication of RT for malignant cases too as a safe approach to achieve successful oncological outcomes.

**FIG. 4.**
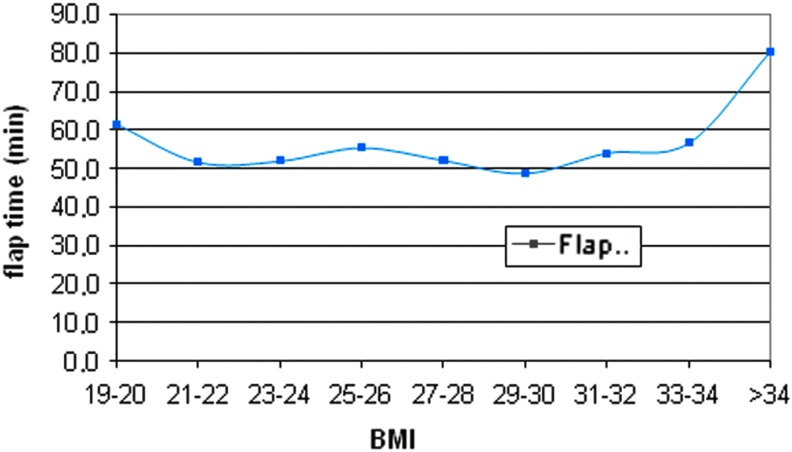
Relation between flap time and BMI. BMI, body mass index.

In consideration of BMI variable, we can assume that in our series we do not find a straight correlation between BMI values and the operation time required; Figure 4 reports that even with increasing BMI values, operative time does not lengthen with a statistical significance (*P* > .05). We also notice that the transaxillary tunnel creation duration (flap time) is not BMI dependent (*P* > .05), demonstrating that endoscopic approach is a safe and feasible technique even for overweight and obese patients. According to these data, overweight and obesity seem to not influence surgical time required, confirming that BMI is not an exclusion criteria for robotic transaxillary thyroidectomy. In our series BMI-range was 17.2–37.5, with a median value similar to the one reported in literature,^25^ whereas in other series BMI reaches 44.5 and, although the procedure would be possible, it may be technically challenging,^26^ but the application of Modena retractor with the endoscopic 30° camera and laparoscopic instruments allows to perform a successful procedure. The introduction of Modena retractor requires a less number of surgeons enrolled to create the transaxillary tunnel and permits an accurate tissue dissection during endoscopic working space creation with human tremors abolition. Modena retractor permits also the utilization of different length blades during the procedure; in our experience a thin shorter blade is used till the strap muscle is lifted up, then a longer one is positioned; the endoscopic view with the use of laparoscopic instruments can avoid a larger blade requirement, even with increasing BMI values.

The drainage debit amount observed for LT and TT was, respectively, inferior to LT+PT and TT+PT one (*P* < .05).

In our series we decided to register and then consider for data analysis first postoperative day serum calcium value: statistical analysis showed that patients who underwent TT and TT+PT operation presented hypocalcemia (with a target laboristic value of 8.5 mg/dL) more frequently than LT and LT+PT groups (*P* < .05); this result can be justified in consideration of the major risk of PTs devascularization or accidental asportation associated to TT surgery. We might expect target PT, independently associated to thyroid surgery both LT and TT, to increase hypocalcemia risk instead of TT operations, but no statistical differences between LT and LT+PT groups. and between TT and TT+PT groups were observed; we can consider this unexpected data as a bias due to the only first postoperative day serum calcium dosage and to the oral calcium support therapy setup starting from the first postoperative day for patients undergoing parathyroid asportation.

In consideration of general postoperative complications reported, we can see that a higher complication rate resulted for patients who underwent longer surgical interventions with *P* < .05; on the contrary, BMI, age, and tumor diameter seem to not influence postoperative complications risk.

In particular, we analyzed the relation between the different BMI categories and some specific postoperative complications: flap hematoma, wound seroma, neck and chest paresthesia, and shoulder discomfort. No significative differences resulted (*P* > .05), demonstrating that overweight and obesity do not increase postoperative complication risk.

Focusing on recurrent laryngeal nerve identification, we know that it could be more difficult in case of Plummer's and Basedow's disease and for malignant lesions, in consideration of fibrosis and anatomically subverted surgical field. In our series the dysphonia risk rate results to increase only in case of malignant nodules (*P* < .05), but the robotic technique, with a 3.78% rate of postoperative dysphonia, confirms to reduce the recurrent laryngeal lesions risk if compared with traditional cervicotomy approach.

Another important aspect, especially when introducing new procedures, is the importance of the surgeon's training and learning curve; in our centre a minimal number of surgeons were enrolled and dedicated to the procedure to progressively reach skills in a step-by-step learning practice program, until they become completely autonomous in performing the whole procedure (first flap and docking time, then console time) and able to manage with the possible complications that may occur. Three were the surgeons involved in this series and all with high experience and skills in advanced endocrine and laparoscopic procedures and robotic abdominal surgery.

Nevertheless, despite the criticisms against RT, such additional costs, longer operative times, and a challenging learning curve,^27,28^ also American Surgeons started to familiarize with robotic thyroid surgery and to approve the procedure for unilateral small nodules only if performed in high-volume thyroid and robotic surgery centres.^22^

According to our experience, a surgical center table to start the transaxillary procedure requires high-volume endocrine surgery and previous good experience in laparoscopic and robotic activity; furthermore, we suggest that a team of at least 3 dedicated surgeons, an initial learning curve performed on lobectomies (almost 20 procedures) before performing total thyroidectomies, strict indications to robotic surgery (20%–25% of total thyroid activity), and well-monitored continuous activity should be required.

## Conclusions

In conclusion, we can assume that this study confirms the application of robotic approach in thyroid surgery as a feasible technique in terms of safety and complications risk; view-magnification, accuracy, easier identification and preservation of both recurrent laryngeal nerve and parathyroid glands, and cosmetic results, represent some of the advantages offered by robotic surgery. The hybrid technique, combined with a dedicated surgical team, can lead to obtain the same outcomes of the traditional anterior cervical approach with advantage of a scarless procedure. With the new advancements in robotic technology more sophisticated tools will be available for endocrine surgeons to obtain progressively more safe and successful outcomes.
